# Efficacy and safety of two Ayurvedic dosage forms for allergic rhinitis: Study protocol for an open-label randomized controlled trial

**DOI:** 10.1186/s13063-019-4004-1

**Published:** 2020-01-07

**Authors:** Jeevani Maheshika Dahanayake, Pathirage Kamal Perera, Priyadarshani Galappaththy, Dulani Samaranayake

**Affiliations:** 1 Dravyaguna Vignanana Unit (Ayurveda Pharmacology and Pharmaceutics), Department of Ayurveda, Institute of Indigenous Medicine, University of Colombo, Colombo, Sri Lanka; 2 Department of Pharmacology, Faculty of Medicine, University of Colombo, Colombo, Sri Lanka; 3 Department of Community Medicine, Faculty of Medicine, University of Colombo, Colombo, Sri Lanka

**Keywords:** Allergic rhinitis, Ayurvedic dosage forms, Tamalakyadi decoctions, Randomized controlled trial

## Abstract

**Background:**

Allergic rhinitis (AR) is an immune response of the nasal mucosa to airborne allergens and involves nasal congestion, watery nasal discharge, itching of the nose, and sneezing. The symptoms of allergic rhinitis may significantly affect a patient’s quality of life and can be associated with conditions such as fatigue, headache, cognitive impairment, and sleep disturbances. Various complementary and alternative medicine treatments have been used for this condition in clinical practice. The Ayurveda system of medicine is the most common complementary medicine system practiced in Sri Lanka. The aim of this study is to examine the efficacy and safety of a decoction used in traditional Ayurveda for allergic rhinitis and its ready- to-use freeze dried formulation in comparison to an antihistamine over a period of 4 weeks on relief of symptoms in allergic rhinitis.

**Study design:**

This is a three-arm, open-label, non-inferiority, randomized controlled clinical trial enrolling patients with AR. Tamalakyadi decoction containing 12 ingredients (TMD12), used in traditional Ayurveda and its freeze-dried formulation are the test products. The efficacy and safety of the two Ayurvedic dosage forms will be tested against the antihistamine loratadine. Patients with symptoms of AR will be allocated randomly into the three arms after a 1-week run-in period and the medications will be given orally for 28 days. Total Nasal symptom Score (TNSS) of the patients will be used as the primary efficacy endpoint. TNSS will be recorded and compared between the three arms prior to visit 1, at the end of 28 days, and end of the first and second months of follow-up. Symptom scores of daytime nasal symptoms, night time nasal symptoms, non-nasal symptoms and health-related quality of life questionnaire are used as secondary end points.

**Discussion:**

This clinical trial will be able to provide evidence-based scientific data on Ayurvedic dosage form, TMD12, and the freeze-dried formulation in the treatment of allergic rhinitis. This trial is expected to develop capacity to scientifically evaluate various Ayurvedic treatments that are claimed to have efficacy in treatment of various disease conditions.

**Trial registration:**

ISRCTN18149439 (6 May 2019).

## Background

Allergic rhinitis is an IgE-mediated immunological response of nasal mucosa to air-borne allergens and is characterized by nasal congestion, watery nasal discharge, itching of the nose, and sneezing. It is commonly defined as seasonal or perennial, depending upon whether symptoms are manifested at defined yearly intervals or throughout the year, respectively [1]. Allergic rhinitis is not life threatening, but for the patient it is an annoying and disturbing disease due to its chronicity and aggravation during exposure to allergic agents. Furthermore, allergic rhinitis is a considerable cause of widespread morbidity, medical treatment costs, reduced work productivity, and lost school days [2]. The symptoms of allergic rhinitis may significantly affect a patient’s quality of life and can be associated with conditions such as fatigue, headache, cognitive impairment, and sleep disturbances [3]. Appropriate management of allergic rhinitis is an important component in effective management of coexisting respiratory conditions such as asthma, sinusitis, and sleep apnea [4].

The World Allergy Organization (WAO) reports the prevalence of rhinitis symptoms in the International Study on Asthma and Allergies in Childhood (ISAAC) to vary between 0.8% and 14.9% in 6 to 7-year-olds and between 1.4% and 39.7% in 13 to 14-year-olds [5]. The reported prevalence in adults ranges from 8.7 to 24.1% in China and 11.4 to 22.7% in Turkey [6]. Also, in the USA, an estimated 20% of cases are seasonal allergic rhinitis and 40% of cases are perennial rhinitis [7].

In Sri Lanka, many people suffer from this condition. A survey of 6000 patients attending the OPD at the Teaching Hospital, Ragama, Sri Lanka, identified allergic manifestations in 8.8% of patients, and 22% of them had rhinitis [8]. Another survey conducted in Sri Lanka among schoolchildren in grade 5 from Western province, Sri Lanka, found symptoms of allergic rhinitis in 21.4% children with a statistically significant higher prevalence in boys [9]. The reason for this observation was not discussed in the paper.

Within Ayurveda and the Sri Lankan traditional medicine system, there are several potentially effective therapeutic methods of treatment for allergic rhinitis, which include internal as well as external treatment methods. Tamalakyadi decoction, which includes 12 ingredients (TMD12), is an herbal decoction used for treatment of allergic rhinitis in Ayurveda [10]. Decoctions are liquid dosage forms prepared freshly from herbs with a 24-h shelf life. Therefore, patients on treatments with decoctions need to prepare it daily, which causes difficulties in their busy lifestyles. Hence, this study plans to develop a ready-to-use dosage form, a freeze-dried powder of TMD12 packed into sachets, and investigate its efficacy and safety. No scientific studies have been published that have evaluated the efficacy and safety of TMD12 decoction either in allergic rhinitis. Therefore this study will evaluate the efficacy and safety of the traditional TMD12 decoction and its freeze-dried formulation in comparison to a non-sedating antihistamine loratadine used in an allopathic system in patients with symptoms of allergic rhinitis.

## Methods

### Study design

This is a three-arm, open-label non-inferiority randomized controlled clinical trial that will be conducted at the National Ayurveda Teaching Hospital in Colombo, Sri Lanka. TMD12, used in traditional Ayurveda, and its freeze-dried formulation will be the test products. The efficacy and safety of the two Ayurvedic dosage forms will be tested against the antihistamine loratadine. Patients with symptoms of AR will be allocated randomly into the three arms after a 1-week run-in period and the medications given orally for 28 days. This study protocol was developed as required by the Standard Protocol Items: Recommendations for Interventional Trials (SPIRIT) (Additional file 1).

Ethics approval has been obtained from Ethics Review Committee, Institute of Indigenous Medicine (ERCIIM), University of Colombo, Sri Lanka (ERC 18/76) and the Ethics Committee of the Faculty of Medicine, University of Colombo, Sri Lanka (EC-18-090). The trial was registered in the ISRCTN registry (Trial number ISRCTN18149439) (Additional file 2).

### Study setting

The study will be conducted in the Ayurveda Teaching Hospital, Borella, and the Institute of Indigenous Medicine (IIM) University of Colombo, Sri Lanka. The study subjects will be recruited from the patients with symptoms of allergic rhinitis who visit to the Outpatient Department (OPD) of Ayurveda Teaching Hospital and those who come, responding to a newspaper advertisement on trial recruitment.

### Participants

Patients will be selected from those seeking treatment for allergic rhinitis. Participation in this research project is voluntary. Patient recruitment is done by screening for eligibility criteria (inclusion and exclusion criteria). Eligible subjects will be randomly assigned to the TMD12 decoction group, TMD12 freeze-dried group (TMD12-FD) or the antihistamine, loratadine group.

### Inclusion and exclusion criteria

The inclusion criteria include (1) age group of 18–65 years at the time of enrollment, of either sex; (2) presence of two or more nasal symptoms (watery rhinorrhea, nasal obstruction, sneezing, and nasal itching); (3) Total Nasal Symptom Score (TNSS) > 6 (0 = no symptoms, 1 = mild symptoms, 2 = moderate symptoms, 3 = severe symptoms); (4) have given written, informed consent to participate in this study.

The exclusion criteria include: (1) Patients with deviated nasal septum/ nasal polyps/ nasal growth/ adenoids/ asthma; (2) Patients with impaired liver and kidney functions, anemia, and unstable cardiovascular conditions or cerebrovascular conditions; (3) currently or previously treated for any malignancy; (4) patients on steroid therapy; (5) already on treatment with TMD12 decoction or antihistamines; (6) pregnant or lactating mothers; (3) those who have known systemic disorders; (4) those who have any history of drug allergy to any of the investigational products; (7) illiterate patients without a literate relative/guardian who can explain the procedures and maintain the patient diary, (8) and any other patients who are considered unsuitable for recruitment by the investigators.

### Sample size

Sample size was calculated based on the primary outcome measurement of TNSS and as for a non-inferiority clinical trial. This study is designed to evaluate the comparative clinical efficacy and safety of two Ayurvedic dosage forms with the antihistamine loratadine, assuming non-inferiority-between the three interventions. Sample size was calculated as specified by Hampel et al. [11]. According to a previous study done among patients with allergic rhinitis using loratadine, clinically significant standardized effect sizes of TNSS are reported in the range of 0.57 to 0.67 [11]. Therefore, a standardized effect size of 0.5 was considered as the non-inferiority margin using the effect reported by Hampel and team.

Sample size was calculated for a significance level (**α**) of 5% and power of 80%. The sample size calculated using these values is 64 per group. With an expected dropout rate of 10%, minimum sample size was calculated as 70 for one arm.

### Recruitment

People who are interested in participating in this clinical study will be provided with a detailed Patient Information Leaflet (PIL) supplemented by verbal explanation of the study procedures. If the participants agree with the information given in PIL, the screening questionnaire will be completed. Informed written consent will be obtained from each participant by the investigators prior to initial interview. The activities in the initial interview will include complete history taking, physical examination, and hematological and biochemical investigations (IgE level, FBS, FBC, ESR, ALT/AST, serum creatinine, UFR). Diagnosis of allergic rhinitis will be made according to the Allergic Rhinitis and its Impact of Asthma (ARIA) criteria. Among them, the participants meeting the inclusion and exclusion criteria will be recruited for the study. All baseline assessment forms (Total Nasal Symptom Score, Quality of life questionnaire and allergic rhinitis symptom score) will be completed by the investigators. Patients will not be allowed to take any other medicines during the trial period. If they have to take any other medicine, they should inform the investigators and discontinue the trial.

### Baseline assessment

Nasal symptoms (watery rhinorrhea, nasal obstruction, sneezing, nasal itching), non-nasal symptoms, HRQoL, hematological and biochemical investigations (IgE level, FBS, FBC, ESR, ALT/AST, serum creatinine, UFR) will be assessed at baseline.

### Randomization

**Fig. 1:**
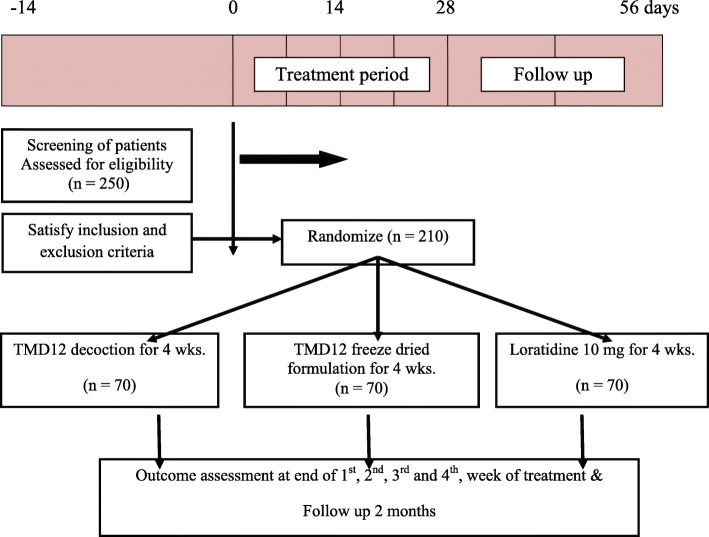
Flow chart of study design

Randomization sequence will be generated using an online randomization website (www.randomisation.com). Block randomization will be done using blocks of 12 to generate the randomization schedule for 240 patients. The patients will be allocated to treatments based on the randomization sequence generated. One week’s supply of the assigned investigational products will be handed over to the patients according to the randomized allocation. Each group will be enrolled with an allocation ratio 1:1:1.The allocation for each randomization number will be put in to individually seal opaque envelops. The envelopes and allocation sequence will be kept under lock and key by one investigator not involved in recruiting patients. The patients meeting inclusion exclusion criteria and recruited into the study will be assigned a randomization number sequentially, according to the date and time of recruitment. The allocated treatments indicated in the sealed envelope for each randomization number will be supplied to each patient. The study design flow chart is shown in Fig. 1.

### Intervention

#### Investigational products

##### Product I - Tamalakyadi decoction (TMD12)

Traditional TMD12 is a brown-colored liquid, prepared using 5 g of 12 plant ingredients: *Phyllanthus niruri* L.*, Terminalia chebula* Retz., *Premna herbacea* Roxb.*, Piper retrofractum*Vahl*, Piper longum* L.*, Solanum trilobatum* L.*, Tinospora cordifolia* (Thunb.) Miers, *Zingiber officinale* Roscoe, *Piper nigrum* L., *Solanum indicum* L.*, Solanum xanthocarpum* L., *Adhatoda vasica* L.The dried plant materials of the 12 ingredients will be ground separately to pre-prepare a coarse powder pack weighing 60 g containing all 12 ingredients. One 60-g pack of this TMD12 dried powder pack will be used to prepare the decoction needed for 1 day. A 1-week supply (seven packs) of this pre-prepared dried herbs pack will be supplied to the patient. They will be informed to put the supplied herbal pack into a pot, add 1920 ml of water and simmer under low flame until the volume is reduced to 240 ml. The process of preparation under standard conditions will be demonstrated to patients who are selected into the TMD12 arm at the Department of Dravyaguna Vignana of IIM using a video. They will be requested to take a daily dose 120 ml twice a day before meals.

##### Product II - Freeze dried Tamalakyadi decoction (TMD12-FD)

This is a freeze-dried powder prepared from 240 ml of TMD12 decoction (TMD 12- FD), which contains 6 g of TMD12-FD packed in triple laminated sachets under a temperature of 19 ^°^C to minimize the moisture absorbance. Preparation will be done at the Research and Development Complex, Herbal Technology Section, Institute of Industrial Technology, Malabe, Sri Lanka under standard laboratory conditions.

The powder should be dissolved in 240 ml of hot water and 120 ml taken twice a day before meals. This reconstituted powder also contains the above-mentioned 12 ingredients in almost the same quantities. In order to develop a ready-to-use formulation of the TMD 12, with composition similar to the TMD12 after preparation, three different formulations were developed and analyzed [12]. These included freeze-dried formulation, spray-dried formulation, and Ganasara formulation and detailed physicochemical and phytochemical analysis were performed. The results are now published and showed that the freeze-dried formulation is quantitatively and qualitatively closest to TMD 12 [12]. Therefore the freeze-dried formulation was selected for evaluation of clinical efficacy and safety in this clinical trial.

##### Product III - Loratadine 10 mg

Non-sedating antihistamine-loratadine 10 mg was selected as the comparator for this clinical trial. Total quantity of loratadine required for the clinical trial, from one of the leading brands of Loratadine will be purchased from one single batch, directly from the importer for the purpose of the trial. The certificate of analysis of the batch will be obtained to check and ensure the quality of the product used. The purchased products will be stored under 25 °C in an air-conditioned environment at the IIM. Patients allocated loratadine arm are requested to take one tablet daily at evening before meals with 240 ml of water. Details of the investigational drugs are shown in Table 1. To minimize the compliance, patients are advised to submit the patient diary and empty containers of the drugs during weekly visits. Also, the day before the clinic date of every week, the chief investigator will be telephoned and send a text message to a mobile phone to be reminded of the clinic date.

**Table 1:** Investigational products

	Drug	Dose	Mode of administration	Route	Method of preparation
1	Tamalakyadi decoction (TMD12)	120 ml	Morning and evening before meals	Oral	60 g (1 packet) of dried powder of plant materials boiled with 1920 ml of water and reduced to 240 ml
2	Freeze dried Tamalakyadi decoction	120 ml	Morning and evening before meals	Oral	6 g (1 sachet) of freeze dried powder dissolve in 240 ml of warm water.
3	Loratadine	10 mg	Evening before meals	Oral	Ingested with 240 ml of water

### Storage, packaging, and dispensing of investigational drugs

All three investigational products (herbal materials of decoction, freeze-dried powder, and loratadine) will be packed for 7 days and labeled, which would indicate the batch number, dose, time of administration, mode of administration. These will be stored in the clinic of the Ayurveda Teaching Hospital, Sri Lanka, to be provided to randomized patients according to the predetermined allocation sequence. A supply of drugs for 7 days will be dispensed to the study participants at weekly visits with instructions.

### Outcome measurements

#### Primary outcome

TNSS of the patients will be used as the primary efficacy endpoint, which has been previously used in allergic rhinitis clinical trials. The mean difference in TNSS will be compared between the three arms at baseline and at the end of 28 days, end of the first month of follow-up and second month of follow-up. The TNSS assesses the symptoms of watery rhinorrhea, nasal obstruction, sneezing, and nasal itching on a four-point scale. The total score range from 0 to 12 where 0 = absent symptoms (no sign/symptom evident), 1 = mild symptoms (signs/symptoms clearly present, but minimal awareness; easily tolerated), 2 = moderate symptoms (definite awareness of signs/symptoms that are bothersome but tolerable), 3 = severe symptoms (signs/symptoms that are hard to tolerate; causes interference with activities of daily living and/or sleep).

#### Secondary outcomes

The following four symptom scores will be used as secondary end points.

1. Mean score of daytime nasal symptoms
2. Mean score of night time nasal symptoms
3. Mean score of non-nasal symptoms
  Patient’s self-rated symptom scores (daily rhinitis diary card) and allergic rhinitis grading symptoms will be used as secondary measures of the efficacy in the clinical trial. Such symptom scores will be collected on a weekly basis during the assessment period. The measurement of symptoms on a four-point rating scale with the following definitions will be used.
  = absent symptoms (no signs/symptoms evident)
  = mild symptoms (signs/symptoms clearly present, but minimal awareness; easily tolerated)
  = moderate symptoms (definite awareness of signs/symptoms that are bothersome but tolerable)
  = severe symptoms (signs/symptoms that are hard to tolerate; causes interference with activities of daily living and/or sleep).
4. Mean score of health-related quality of life score – Health-related quality of life is measured using allergic rhinitis symptoms at the baseline and end of the intervention (after 4 weeks, 1 month of follow-up, and 2 months of follow-up).

Changes in the serum IgE level and eosinophil count will be studied by comparing before and after treatment values. Procedures related to the study are shown in Table 2.

**Table 2:**
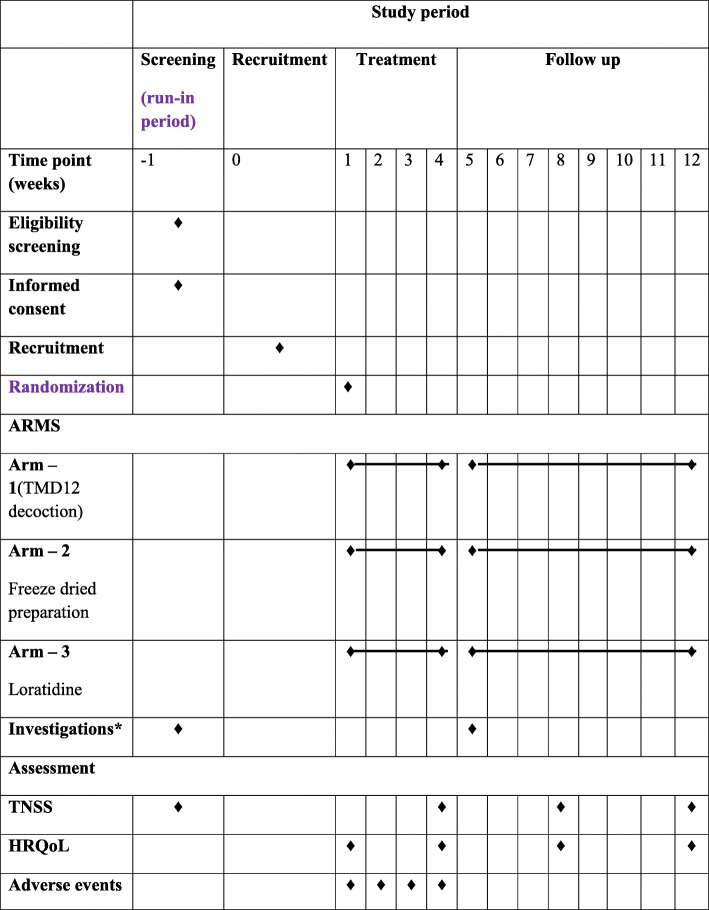
Study procedures

#### Safety assessment

Each patient will undergo hematological and biochemical investigations (FBS, FBC, ESR, AST/ALT, and serum creatinine/GFR), urine full report before and after the interventions, which are done primary for safety assessment.

All adverse events experienced by patients will be recorded weekly by the investigators at every visit to the clinic. Further, patients will be advised to record any adverse reactions in their patient diaries and will be advised to inform the investigators using the given contact numbers. They will also be advised to come to the clinic for assessment when they have any unexpected symptoms or complaints. If any serious adverse events occur, they will be carefully assessed and reported to the ERC of IIM and Medical Faculty within 5 working days. No serious adverse reactions are expected with any of the three study medications. However, in the events of an adverse reaction requiring in-hospital management, the facilities and expert management would be provided and the complete clinical trial will be terminated prematurely if there is evidence that the safety of the trial participants can no longer be assured or new scientific information arises during the course of the clinical trial regarding patient safety.

### Data handling, record keeping, and dissemination

An individual file for each participant will be used to archive a hard copy of the case record forms including informed consent, results of the hematological and biochemical investigations, results of the physical examinations, and completed questionnaires. Data will be entered by a minimum number of dedicated staff and saved in a dedicated computer with password protection. These data will be retained with the researchers and will not be handed over to any other party under any circumstances. The study participant’s information will be securely stored at each clinic visit during the study. At the end of the study, all records will continue to be kept in a secure location for a 2-year period.

Study participants’ data will be stored at the department of Dravyaguna Vignana, IIM, which will be used for statistical analysis and scientific reporting. Each participant’s contact or identifying information will be separately stored. Individual participants and their research data will be identified by a unique study identification number. At the end of the study, all study databases will be de-identified and archived. A data safety monitoring board has been appointed according to the guidelines of Ethics Review Committee of Faculty of Medicine for safety monitoring. The board consists of three independent expert members. Further, we have not planned the auditing of this clinical trial, because it is a single-center trial involving only 210 patients. The results of the study will be used for scientific reporting in conferences and will be published in peer-reviewed journals. Further results of the study and the grouping information of the participants will be provided to the individual after completion of the trial.

### Ethical considerations

The approval of the research protocol has been obtained from the Research approval committee of the Faculty of Graduate Studies, University of Colombo and the Ethics Review Committees of Institute of Indigenous Medicine (IIM) and Faculty of Medicine, University of Colombo. The trial was registered in ISRCTN registry (Trial number ISRCTN18149439 10.1186/ISRCTN18149439). The study will be conducted adhering to Good Clinical Practice (GCP) guidelines. Protocol modifications will be informed to Ethics Review Committees and the trial registry for their approval.

Patients will be provided with an information sheet with the details of the research given in all three languages (Sinhala/Tamil/English) and written consent will be obtained before participation. The information provided will include the nature, duration, and possible consequences of the trial. A patient may withdraw his or her consent to participate in this study at any time, with no penalty or effect on medical care or loss of benefits. The questionnaire will be interviewer-administrated and anonymous. A minimal amount of data needed to assess the socio demographic data will be gathered. This will include occupation and nature of health condition. Researchers will not collect any other personal data.

## Method of data analysis

For primary and secondary outcome measures, the mean values at baseline and at the end of the study and the mean differences will be compared between the three arms using ANOVA (analysis of variance) or the non-parametric Kruskal–Wallis test, depending on the normality of the data. Within each treatment arm, the difference in primary and secondary outcome measures before and after the intervention period will be compared using paired-sample *t* test or non-parametric Wilcoxon signed-rank test, depending on the normality of the data. 95% confidence intervals will be calculated for all outcome measures that are normally distributed. Categorical variables will be compared between groups using the Chi-square test. Possible confounders will be adjusted using ANCOVA. Following adjustment for confounding using ANCOVA, adjusted mean values with confidence intervals will be calculated and reported. Statistical analysis will be performed using the SPSS Statistical Package program (ver. 18.0), and the level of significance will be established at α = 0.05. Missing data at outcome assessment will be replaced with the available latest values of the outcome measure. Intention-to-treat analysis will be performed for all efficacy outcomes and safety outcomes. In addition, per-protocol analysis will be performed for the efficacy outcomes by including only the patients completing the follow-up.

## Discussion

Allergic rhinitis is a chronic respiratory disease with significant health and psychological burden to patients due to the prolonged disease course [13]. In Sri Lanka, also several people seeking treatments for allergic rhinitis from Ayurvedic hospitals. The symptoms of allergic rhinitis may significantly affect a patient’s quality of life and can be associated with conditions such as fatigue, headache, cognitive impairment, and sleep disturbances. Previous studies recognize findings introduce it as a major risk factor for developing asthma and other respiratory disorders [14]. Allergic rhinitis imposes a great financial burden on both the individual and society due to health care and social costs associated with the disease [15]. Therefore, effective treatment would be important for treating this disease. At present, pharmacotherapies consists of oral and intranasal antihistamines, mast cell stabilizers, decongestants, intranasal steroids, leukotriene inhibitors, and allergy immunotherapy [16]. Due to fear of side effects and adverse effects of allopathic drugs some patients prefer to use herbal remedies for their ailments in Sri Lanka. From ancient times until now, many Sri Lankan people are willing to get traditional herbal treatments for their ailments.

Tamalakyadi decoction is an herbal decoction that has been prescribed for allergic rhinitis in Ayurveda and Sri Lankan traditional systems of medicine for a long time. Decoctions are liquid dosage forms that have to be prepared daily due to their short shelf life. Hence in this study an attempt was made to prepare a ready-to-use modified dosage form from Tamalakyadi decoction, produced as a freeze-dried powder.

Our research team designed this three-arm, open-label, non-inferiority randomized control trial to compare and evaluate the effectiveness of TMD12 and its freeze-dried formulation in allergic rhinitis compared to an antihistamine used in an allopathic system of medicine. This clinical trial will be able provide evidence-based scientific data on the classical Ayurvedic dosage form TMD12 and the new dosage form, the freeze-dried powder in the treatment of allergic rhinitis. This trial is also expected to develop the capacity to scientifically evaluate Ayurvedic treatments in Sri Lanka that could help patients having chronic conditions such as allergic rhinitis.

### Strengths and limitations

To our knowledge, this is the first randomized controlled clinical trial to investigate the efficacy of an herbal decoction with its modified dosage form in Sri Lanka for allergic rhinitis. Results of this study will provide evidence regarding the use of this herbal preparation for the treatment of allergic rhinitis.

## Trial status

This protocol is version 1. The recruitment is currently in progress and it is expected that the recruitment will be completed by the end of May 2021.

## Supplementary information

**Additional file 1.** SPIRIT 2013 Checklist: Recommended items to address in a clinical trial protocol and related documents.

**Additional file 2.** Clinical Trial registry: ISRCTN18149439 https://doi.org/10.1186/ISRCTN18149439.

**Additional file 3.** Informed consent form.

## Data Availability

Not applicable. This manuscript is a protocol for a randomized clinical trial and does not contain any data.
