# Supplementary material for: Efficacy and safety of two Ayurvedic dosage forms for allergic rhinitis: Study protocol for an open-label randomized controlled trial
Source: Trials. 2020 Jan 7;21:37. doi: 10.1186/s13063-019-4004-1 (PMC6947868; doi:10.1186/s13063-019-4004-1)
Supplement: Supplementary file 2 — Additional file 2. Clinical Trial registry: ISRCTN18149439 https://doi.org/10.1186/ISRCTN18149439. [file 13063_2019_4004_MOESM2_ESM.pdf]

ISRCTN registry
ISRCTN18149439 https://doi.org/10.1186/ISRCTN18149439 [https://doi.org/10.1186/ISRCTN18149439]

Comparing the effect of two Ayurveda drugs on the treatment of allergic rhinitis

[#]

Condition category
Respiratory
Date applied
18/04/2019
Date assigned
02/05/2019
Last edited
10/05/2019

Prospective/Retrospective
Prospectively registered
Overall trial status
Ongoing
Recruitment status
Recruiting

Plain English Summary

Background and study aims
Allergic rhinitis is an immune response of the nasal mucosa (lining of the nasal cavities) to airborne allergens and involves nasal congestion, watery nasal discharge, itching of the nose and sneezing. Allergic rhinitis is commonly defined as seasonal or perennial, depending upon whether symptoms occur at defined yearly intervals or throughout the year, respectively. Allergic rhinitis is not life threatening, but it is an annoying and disturbing disease for the patient due to its chronicity and aggravation when exposed to allergic agents. Furthermore allergic rhinitis is a considerable cause of widespread morbidity, medical treatment costs, reduced work productivity and lost school days. The symptoms of allergic rhinitis may significantly affect a patient's quality of life and can be associated with conditions such as fatigue, headache, cognitive impairment and sleep disturbances. Appropriate management of allergic rhinitis is an important component in the effective management of coexisting or complicated respiratory conditions such as asthma, sinusitis and sleep apnea. In this context, various complementary and alternative medicine treatments have been used for this condition in clinical practice. The Ayurveda system of medicine is the most common complementary medicine system in Sri Lanka. The aim of this study is to find out whether the use of two preparations (decoction and its freeze dried powder) over a period of 4 weeks is able to cure the symptoms of allergic rhinitis.

Who can participate?
Male and female patients aged 18 to 65 with allergic rhinitis

What does the study involve?
Participants are randomly allocated to receive either one of two Ayurveda treatments (traditional herbal decoction or a sachet containing freeze dried ingredients of herbal decoction) or the antihistamine loratidine for 4 weeks. Nasal symptoms are assessed at the start of the study, after four weeks of treatment, and after one month and two months of follow up.

What were the possible benefits and risks of participating?
The participants receive information and advice from a specialized medical team. In addition their participation may help to develop an Ayurveda drug treatment for allergic rhinitis.

Where is the study run from?
National Ayurveda Teaching Hospital (Sri Lanka)

When is the study starting and how long is it expected to run for?
January 2019 to May 2021

Who is funding the study?
University Grants Commission (Sri Lanka)

Who is the main contact?
Dr Jeevani Dahanayake
jeevanimd@iim.cmb.ac.lk

Trial website

[]

Contact information

Type

Scientific

Primary contact

Dr Jeevani Dahanayake

ORCID ID

http://orcid.org/0000-0002-9085-918X [http://orcid.org/0000-0002-9085-918X]

Contact details

Institute of Indigenous Medicine
University of Colombo
Sri Lanka
Colombo
0094
Sri Lanka
+94 (0)772961461
jeevanimd@iim.cmb.ac.lk [mailto:jeevanimd@iim.cmb.ac.lk]

Type

Public

Additional contact

Dr Pathirage Kamal Perera

ORCID ID

http://orcid.org/0000-0003-0337-1336 [http://orcid.org/0000-0003-0337-1336]

Contact details

Institute of Indigenous Medicine
University of Colombo

Colombo
0094
Sri Lanka
+94 (0)716419072
kamalperera@iim.cmb.ac.lk [mailto:kamalperera@iim.cmb.ac.lk]

Type

Scientific

Additional contact

Prof Priyadarshani Galappatty

ORCID ID

http://orcid.org/0000-0002-9279-9053 [http://orcid.org/0000-0002-9279-9053]

Contact details

Department of Pharmacology
Faculty of Medicine
University of Colombo
Colombo
0094
Sri Lanka
+94 (0)718655651
p.galappatthy@pharm.cmb.ac.lk [mailto:p.galappatthy@pharm.cmb.ac.lk]

Additional identifiers

EudraCT number

Nil known

ClinicalTrials.gov number

Nil known

Protocol/serial number

2016/Mphil-PhD/029

Study information

Scientific title

Development of an Ayurvedic pharmaceutical preparation for allergic rhinitis and evaluation of its safety and efficacy

Acronym

AyudrugAR

Study hypothesis

Freeze dried powder of Tamalakyadi decoction will have similar efficacy and safety in treating patients with allergic rhinitis compared to the traditional decoction and antihistamines.

Ethics approval

Approved 22/01/2019, Ethics Review Committee (Institute of Indigenous Medicine, University of Colombo, Rajagiriya, Sri Lanka; Tel: +94 (0)112692395; Email: ethicsreviewiim@gmail.com), ref: ERC 18/76

Study design

Three-arm open-label non-inferiority randomized control clinical trial

Primary study design

Interventional

Secondary study design

Randomised controlled trial

Trial setting

Hospitals

Trial type

Treatment

Patient information sheet

Not available in wed format, please use contact details to request a participant information sheet.

Condition

Allergic rhinitis

Intervention

This will be a three-arm open-label non-inferiority randomized control trial in patients with allergic rhinitis. Consecutive consenting sample method will be followed to select participants of the arms for the study. A blocked design will be used, using an online statistical computing web programming to generate the randomization schedule (research randomizer https://www.randomizer.org [https://www.randomizer.org]). Eligible subjects will be randomly assigned to Arm I, Arm II and Arm III to receive herbal decoction, freeze-dried powder of herbal decoction and antihistamine for 28 days.

The patients of Arm I will be treated with herbal decoction (Tamalakyadi decoction), 120 ml twice a day after meals. Arm II patients will be treated with 6 g of freeze-dried powder of herbal decoction. The powder should be dissolved in 240 ml of hot water and should take 120 ml twice a day after meals. The patients of Arm III will receive antihistamine (loratidine 10 mg) at night taken with 240 ml of water. Patients belong to three arms have to visit the clinic weekly.

Total Nasal Symptom Score (TNSS) and IgE level of patients will be the primary efficacy endpoints. The mean difference in TNSS and IgE level will be compared between the three arms as the primary endpoints at the end of 28 days. The TNSS will be again analyzed after 1 month and 2 months of treatment at follow-up visits.

Mean score of daytime nasal symptoms, nighttime nasal symptom, non-nasal symptoms and Health-Related Quality of Life score will be used as secondary endpoints in the clinical trial. These symptom scores will be

analyzed by using the information mentioned in rhinitis diary card of the patient. This diary cards will be collected weekly at the clinic.

Follow up – patients will be assessed after 1 month and 2 months at the clinic without a drug intervention.

Intervention type

Drug

Phase

Phase II

Drug names

Herbal decoction of Tamalakyadi decoction, freeze-dried powder of Tamalakyadi decoction, antihistamine (loratidine)

Primary outcome measure

1. Nasal symptoms (watery rhinorrhea, sneezing, nasal obstruction, nasal itching) measured using the Total Nasal Symptom Score (TNSS) of allergic rhinitis patients at baseline and the end of intervention (after four weeks, after one month of follow up and two months of follow up)
2. Serum Immunoglobulin E level measured using chemiluminesent enzyme immunoassay (EIA) at baseline and after intervention

Secondary outcome measures

1. Mean score of daytime nasal symptom score
2. Mean score of nighttime nasal symptom score
3. Mean score of non-nasal symptoms
Patient self-rated symptom scores (daily rhinitis diary card) and allergic rhinitis grading symptoms collected on a weekly basis during the assessment period. The measurement of symptoms on a 4-point rating scale with the following definition will be used:
• 0 = absent symptoms (no sign/symptom evident)
• 1 = mild symptoms (sign/symptom clearly present, but minimal awareness; easily tolerated)
• 2 = moderate symptoms (definite awareness of sign/symptom that is bothersome but tolerable)
• 3 = severe symptoms (sign/symptom that is hard to tolerate; causes interference with activities of daily living and/or sleeping)
4. Health-related quality of life measured using HRQoL questionnaire (Valero et al, 2009 & 2013) at baseline and end of intervention (after four weeks, one month of follow up and two months of follow up)

Overall trial start date

22/01/2019

Overall trial end date

01/05/2021

Reason abandoned (if study stopped)

Eligibility

Participant inclusion criteria

1. Age 18-65 years at the time of enrollment, of either sex
2. No known systemic disorders
3. Newly diagnosed allergic rhinitis patients on Ayurvedic treatment
4. No history of drug allergy
5. Non-pregnant and non-breastfeeding
6. Have given written informed consent to participate in this study
7. Total Nasal Symptom Score (TNSS) more than 6

Participant type

Patient

Age group

Adult

Gender

Both

Target number of participants

70 patients for each arm

Participant exclusion criteria

1. Patients with deviated nasal septum/nasal polyps/nasal growth/adenoids/asthma
2. Patients with impaired liver and kidney functions, anaemia, and unstable cardiovascular conditions or cerebrovascular conditions
3. Currently or previously treated for any malignancy
4. Patients on steroid therapy
5. Already on treatment with herbal decoction or antihistamines
6. Pregnant or lactating mothers
7. Illiterate patients without a literate relative/guardian who can explain the procedures and maintain the patient diary

Recruitment start date

30/05/2019

Recruitment end date

30/05/2021

Locations

Countries of recruitment

Sri Lanka

Trial participating centre

National Ayurveda Teaching Hospital
Dr. N. M. Perera Mawatha, Borella, Colombo 8
Colombo

0094
Sri Lanka

Sponsor information

Organisation

University of Colombo

Sponsor details

Faculty of Graduate Studies 35/30
Prof Stanley Wijesundera Mawatha
Colombo 7
Colombo
0094
Sri Lanka
+94 (0)11 205 56 56
office@fgs.cmb.ac.lk [mailto:office@fgs.cmb.ac.lk]

Sponsor type

University/education

Website

http://fgs.cmb.ac.lk [http://fgs.cmb.ac.lk]

Funders

Funder type

Government

Funder name

University Grants Commission - Sri Lanka

Alternative name(s)

UGC

Funding Body Type

government organisation

Funding Body Subtype

Federal/National Government

Location

Sri Lanka

Results and Publications

Publication and dissemination plan

Planned publication in high impact peer-reviewed journals.

Additional documents will be available upon request from Dr Jeevani Dahanayake (jeevanimd@iim.cmb.ac.lk). Study protocol planned to published in a peer-reviewed indexed journal.

IPD sharing statement
The datasets generated during and/or analysed during the current study are/will be available upon request from Dr Jeevani Dahanayake (jeevanimd@iim.cmb.ac.lk). Study participant data sheets will not include contact or identifying details. Study data entry and study management systems used by clinical sites will be secured and password protected. At the end of the study, all study databases will be de-identified and archived. Availability of raw data of the study is based on the above conditions.

Intention to publish date

01/05/2021

Participant level data

Available on request

Basic results (scientific)

Publication list

Publication citations

Additional files

Editorial Notes

10/05/2019: The following changes have been made: 1. The recruitment start date has been changed from 06/05/2019 to 30/05/2019. 2. The recruitment end date has been changed from 15/04/2021 to 30/05/2021.
24/04/2019: Trial's existence confirmed by Ethics Review Committee.
