# Supplementary material for: Efficacy and safety of two Ayurvedic dosage forms for allergic rhinitis: Study protocol for an open-label randomized controlled trial
Source: Trials. 2020 Jan 7;21:37. doi: 10.1186/s13063-019-4004-1 (PMC6947868; doi:10.1186/s13063-019-4004-1)
Supplement: Supplementary file 3 — Additional file 3. Informed consent form. [file 13063_2019_4004_MOESM3_ESM.docx]

**Development of an Ayurvedic pharmaceutical preparation for allergic rhinitis and evaluation of its safety and efficacy**

**INFORMATION SHEET**

I am Dr. JeevaniMaheshikaDahanayake, attached to the Institute of Indigenous medicine. My current designation is Senior Lecturer. I would like to invite you to take part in the research study onDevelopment of aready to use formulation of an Ayurvedic decoction for allergic rhinitis and evaluation of its safety and efficacy conducted by Prof. Priyadarshani Galappatty, Dr. P.K. perera and myself atBorellaAyurveda Teaching Hospital.

1. **Purpose of the study**

The purpose of this research is toassess the efficacy and safety of a ready to use formulation of an Ayurvedicherbal decoction developed as a powderon patients with Allergic rhinitis.

1. **Voluntary participation**

Your participation in this study is voluntary. You are free to not participate at all or to withdraw from the study at any time despite consenting to take part earlier. There will be no loss of medical care or any other available treatment for your illness or condition to which you are otherwise entitled. If you decide not to participate or withdraw from the study you may do so at any time.

1. **Duration, procedures of the study and participant’s responsibilities**

This study will be conducted over a period of one month. If you volunteer to participate in this study, we will ask you to do the following:

You will be provided with either **(a)7 packets to prepare decoction at home or (b) 7 decoction sachets to prepare decoction at home or (c) western medicine called Loratidine** at weekly intervals for 28 days.

If you are selected to **prepare decoction**

1. You will need to prepare the decoction by adding the content of the packet into a earthen pot or stainless steel vessel with 1920 ml (8 patha) water and heat in mild fire and reduce to 240 ml (1 patha). Then consume half the volume (120 ml) before breakfast and the other half (120 ml) before dinner, around 6 am in the morning and 6 pm in the evening.

**(A measuring cup will be provided to measure water and prepared decoction)**

If you are selected to **decoction sachets**

1. You will need to prepare the decoction by adding the content of the sachet into 240 ml of hot water and dissolve and consume half the volume (120 ml) before breakfast and the other half (120 ml) before dinner, around 6 am in the morning and 6 pm in the evening.

**(A measuring cup will be provided to measure water and prepared decoction)**

If you are selected to **Loratidine**

1. You will need to take 1 tablet after meal in the evening.

The decoction / tablet should be drunk continuously for 28 days. After the treatment you have to visit our clinic at monthly intervals for 2 months to asses you disease condition further.

You will be asked to come on Day 0, 8^th^, 15^th^, 22^nd^and 29^th^ to the hospital to assess your health conditions and improvement. On these days a questionnaire will be administered to you by the investigators to collect data on your views regarding these drugs. **You will be undergo hematological and biochemical investigations. For that purpose b**efore starting the treatment and 29^th^ day of the treatment period, blood (5 ml) and urine samples will be taken for the analysis by a qualified Phlebotomist (Nurse/MLT).

We will give you a booklet named as “Diary”. You have to mention your daily symptoms related to the allergic rhinitis in the booklet. Detailed information will give at the 1^st^ visit (day 0) to fill the diary properly.

1. **Potential benefits**

Participation in this study may benefit you by your contribution to a new therapy gaining access to scientifically proved treatment which is being used in Ayurveda; you will be playing an active role in your own health care and helping others by contributing to this medical research.

1. **Risks, hazards and discomforts**

As blood will be drawn, there will be physical discomfort to the participants.

1. **Reimbursements**

You will not be paid for your participation in this research since funds have not been allocated for this purpose in the grant, but the transport and the treatment costs will be borne by the investigator.

1. **Confidentiality**

Confidentiality of all records is guaranteed and no information by which you can be identified will be released or published. These data will never be used in such a way that you could be identified in any way in any public presentation or publication without your express permission.

1. **Termination of study participation**

You may stop participating in this study at any time (with no penalty or effect on medical care or loss of benefits). Please notify the investigator as soon as you decide to withdraw your consent.

1. **Clarifications**

If you have questions about any of the tests / procedures or information please feel free to ask any of the persons listed below.

Dr. JeevaniDahanayake (071-5347043)

Dr. P.K. Perera (071-6419072)

Prof. PriyadarshaniGalappatty (0718655651)

**CONSENT FORM**

**Part A - To be filled by the participant**

The participant should complete the whole of this sheet herself.

1.Have you read the information sheet? (Please keep a copy for yourself) YES/NO

2.Have you had an opportunity to discuss this study and ask any questions? YES/NO

3.Have you had satisfactory answers to all your questions? YES/NO

4.Have you received enough information about the study? YES/NO

5.Who explained the study to you?

………………………………………………………………..

6.Do you understand that you are free to withdraw from the study at any time, without having to give a reason and without affecting your future medical care? YES/NO

7.Information held by the investigators relating to your participation in this study may be examined by other research assistants. All personal details will be treated as STRICTLY CONFIDENTIAL. Do you give your permission for these individuals to have access to your records? YES/NO

8.Have you had sufficient time to come to your decision? YES/NO

9.Do you agree to take part in this study? YES/NO

Participant’s signature: …………………………..…………

Date:………………………………….

Name (BLOCK CAPITALS):

…………………………………………………………………………………………………………………………………………………………………………………………………….

**Part B - To be filled by the investigator**

I have explained the study to the above volunteer and she has indicated her willingness to take part.

Signature of investigator: ……………………................

Date: ……………………………………………………………….

Name (BLOCK CAPITALS):

………………………………………………………………………………………………
